# Experimental and Mathematical Studies on the Drug Release Properties of Aspirin Loaded Chitosan Nanoparticles

**DOI:** 10.1155/2014/613619

**Published:** 2014-06-01

**Authors:** Yixiang Shi, Ajun Wan, Yifei Shi, Yueyue Zhang, Yupeng Chen

**Affiliations:** ^1^School of Chemistry and Chemical Engineering, Shanghai Jiaotong University, 800 Dongchuan Road, Shanghai 200240, China; ^2^State Key Laboratory of Pollution Control and Resources Reuse, College of Environmental Science and Engineering, Tongji University, Shanghai, China; ^3^School of Life Sciences and Biotechnology, Shanghai Jiaotong University, 800 Dongchuan Road, Shanghai 200240, China

## Abstract

The study of drug release dynamic is aiming at understanding the process that drugs release in human body and its dynamic characteristics. It is of great significance since these characteristics are closely related to the dose, dosage form, and effect of the drugs. The Noyes-Whitney function is used to represent how the solid material is dissolved into solution, and it is well used in study of drug dynamic. In this research, aspirin (acetylsalicylic acid (ASA)) has been encapsulated with different grades of chitosan (CS) varying in molecular weight (Mw) for the purpose of controlled release. The encapsulation was accomplished by ionic gelation technology based on assembly of positively charged chitosan and negatively charged sodium tripolyphosphate (TPP). The encapsulation efficiency, loading capacity, and drug release behavior of aspirin loaded chitosan nanoparticles (CS-NPs) were studied. It was found that the concentration of TPP and Aspirin, molecular weights of chitosan have important effect on the drug release patterns from chitosan nanoparticles. The results for simulation studies show that the Noyes-Whitney equation can be successfully used to interpret the drug release characteristics reflected by our experimental data.

## 1. Introduction

Percutaneous transluminal coronary angioplasty (PTCA) is an accepted treatment for providing relief of angina pectoris in patients with single- and multivessel disease [1, 2]. Coronary restenosis remains the primary limitation of PTCA [3, 4]. Antiplatelet therapy with acetylsalicylic acid (aspirin) is well established as secondary prophylaxis in patients with arterial thrombotic disorders [5, 6]. Studies have also demonstrated the prophylactic effect of low-dose aspirin on reducing the risk of future cardiovascular events in a variety of clinical settings [7]. Aspirin (ASA) is an anti-inflammatory pain killer, which is one of the hydrophilic drugs. It is extensively used for pain relief, to reduce inflammation and temperatures and to reduce the risk of heart attacks and strokes [8].

Drug delivery systems (DDS) such as lipid- or polymer-based nanoparticles can be designed to improve the pharmacological and therapeutic properties of drugs administered parenterally [9]. Chitosan is a naturally occurring biopolymer made up of *β*-(1,4)-linked glucosamine units [10, 11]. It is produced by deacetylation of chitin extracted from shells of crabs, shrimps, and krills [12]. Being versatile due to its chemical structure, chitosan has received increasing attention as a renewable polymeric material. Biodegradability, low toxicity, and good biocompatibility make chitosan particularly suitable for use in biomedical and pharmaceutical formulations [13–15]. It has shown excellent properties as excipient and has been used as a vehicle in compressed tablets, as a disintegrant, as a binder, and as a granulating agent in ground mixtures, as well as a cogrinding diluent to boost dissolution rate and bioavailability of water-insoluble drugs [16–18]. Chitosan has also been widely investigated in the development of controlled release drug delivery systems [19–27]. Particularly, it has mucoadhesive properties due to its positive charge which gives it electrostatic interaction with the negatively charged mucosal surface, making drugs easier to absorb transmucosally.

Although there has been considerable interest in developing chitosan nanoparticles (NPs) as effective drug delivery devices [6, 13, 28], a majority of these studies have dealt with the preparation method of drug loaded CS-NPs. Up to date, the study of drug loaded chitosan nanoparticles was focused on release behavior of the drug from chitosan matrix nanoparticles. Depending on the concentration, deacetylation degree and Mw of chitosan, as well as the concentration of sodium tripolyphosphate, the liberation of drug from the chitosan nanoparticles varied from fast release to slow release [29–31]. Therefore, it is of great interest to investigate the interaction mechanism of the factors which influenced the release behavior of drug from CS-NPs.

Pharmacokinetics is the study focusing on the release profiles of drugs under different conditions. Its most immediate application is to study the drug release process within human body. By pharmacokinetic studies, we can determine the best way for drug administration, what is the suitable dosage form, how many drugs per dose, how many times the drug needed to be given each day, and its efficacy. Therefore, it is important to set the right kinetic parameters for drug release. Based on the experimental data of kinetic curves, we may sum up the mathematical model for the drug release process and thus be able to provide guidance for future experiments.

In this study, the chitosan nanoparticles (CS-NPs) were obtained by ionic gelation technology based on assembly of positively charged chitosan and negatively charged sodium tripolyphosphate (TPP). With aspirin (ASA) as the model drug, the encapsulation efficiency, loading capacity, and drug release behavior in vitro were studied. Morphology of the prepared aspirin CS-NPs was observed by transmission electron microscopy (TEM). The characterization and thermal behavior of aspirin in CS-NPs were studied by the Fourier transform infrared (FT-IR) spectroscopy and differential scanning calorimetry (DSC). The effect of the concentration of TPP and ASA and molecular weight of chitosan were discussed in detail. In our computational simulations, we used and tested three different mathematical models, which are probabilistic Boolean network model, the model based on polynomial fitting, and the differential equation based on the Noyes-Whitney equation [32].

## 2. Materials and Methods

### 2.1. Materials

Chitosan with different molecular weight (Mw: 210 KDa and 670 KDa; the degree of deacetylation was 90%) was obtained from Dacheng Biotech. Co. Ltd. (Weifang, China). Aspirin was obtained from Lunan Pharmaceutical Co. Ltd. (Linyi, China). Sodium tripolyphosphate (TPP) and other reagents were all analytical reagents grade. Unless otherwise stated, the chitosan used in this research is mainly 210 KDa.

### 2.2. Preparation of Aspirin Loaded CS-NPs

Chitosan with different Mw was dissolved in 1% (v/v) acidic aqueous solution at the concentration of 2 mg/mL. 10 mg, 15 mg, 25 mg, and 30 mg of aspirin were added to the 25 mL chitosan acidic solution. After dissolving completely, Tween-80 (2% v/v) was added as a surfactant by using homogenizer at 500 rad/s for 20 min. 10 mL TPP solution with the different mass concentration (2.5 mg/mL; 3.0 mg/mL; 4.0 mg/mL; 5.0 mg/mL) was added into the preformed aspirin chitosan solutions separately. After cross-linking for 60 min, the aspirin loaded CS-NPs were washed with distilled water repeatedly and then freeze-dried.

### 2.3. The Fourier Transform Infrared (FT-IR) Spectroscopy

Transmission infrared spectra of CS-NPs loaded with various concentrations of aspirin, pure aspirin, and pure chitosan were measured by using a Fourier transform infrared spectrophotometer 430 (PE, USA). About 2 mg of various samples was mixed with 100 mg KBr, and prepared pellets were used for studies.

### 2.4. Differential Scanning Calorimetry (DSC)

The DSC thermograms of pure aspirin and aspirin loaded CS-NPs were prepared in the condition of 0.8 mg/mL aspirin and 2.5, 3.0, and 4.0 mg/mL TPP were recorded with a heating rate of 20°C/min in nitrogen between 25°C and 180°C.

### 2.5. Morphology Study

The surface morphology of CS-NPs was observed by transmission electron microscopy (TEM). The CS-NPs solution was dropped on copper grids and dried overnight at room temperature for viewing.

### 2.6. Evaluation of Drug Loaded Efficiency

Aspirin encapsulation efficiency and loading capacity were studied by separating CS-NPs from the aqueous medium containing free drug by ultracentrifugation with 9,000 rpm at room temperature for 30 min. The amount of free aspirin was determined by UV spectrophotometer at 298 nm. Dilutions of samples and calibration curves were performed in phosphate buffered saline (pH 7.4). The aspirin encapsulation efficiency (EE) and the aspirin loading capacity (LC) of the process were calculated from (1) and (2) indicated below:

(1) EE=(total  amount  of  aspirin−free  aspirin)total  amount  of  aspirin×100%

(2) LC=(total  amount  of  aspirin−free  aspirin)nanoparticles  weight×100%.

### 2.7. In Vitro Drug Release Study

The CS-NPs were collected by ultracentrifugation at 9,000 rpm for 30 min. They were dispersed in phosphate buffered saline (pH 7.4) at 37°C under magnetic stirring. At various time points, supernatants were isolated by ultracentrifugation, and 3 mL sample was taken out instead of the same volume of fresh medium. The amount of released aspirin was analyzed with UV spectrophotometer (PE, USA) at 298 nm.

### 2.8. Mathematical Fitting Using the Noyes-Whitney Equation

The Noyes-Whitney equation was developed by some physical chemists at the beginning of the 20th century to characterize of the process of solid dissolution [33, 34]. The equation is

(3) dCdt=DSVh(Cs−C),

where *C* is the concentration of a substance in the solution, *t* is time, *dC*/*dt* is the rate of dissolution, *C*_*s*_ is the solubility of the substance in the solvent, *D* is the diffusion coefficient, *S* is the surface area of the solid, *V* is the volume of the medium, *h* is the thickness of the diffusion layer. Since *D*, *V*, and *h* are all constants, usually *K* is used in the place of *D*/(*V*∗*h*) as a constant. Therefore, it is possible for us to know how the concentration will change through time by solving this differential equation.

The key for using the Noyes-Whitney fitting is to solve the differential equation. Since the dissolution rate is equal to the derivative of drug concentration over time, we can reverse this operation to get the relationship between the concentrations versus time. First we combine the constants in the formula and get *dC*/*dt* = *P*(*C*_*s*_ − *C*), as *P* = (*DS*)/(*Vh*), so

(4) $$\begin{array}{c} \frac{dC}{\left(C_{s}-C\right)}=P*dt. \end{array}$$

Solving this differential equation, we get −ln⁡(*C*_*s*_ − *C*) = *Pt* + *b*, *b* is a constant so

(5) $$\begin{array}{c} C_{s}-C=e^{\left(-(Pt+b)\right)}\\ C=C_{s}-e^{\left(-(Pt+b)\right)}. \end{array}$$

When *t* = 0, *C* will be 0 too, so we have

(6) $$\begin{array}{c} C_{s}=e^{\left(-b\right)}\\ b=-\ln\left(C_{s}\right). \end{array}$$

Then the final equation is

(7) $$\begin{array}{c} C=C_{s}-e^{\left(-Pt\right)}*C_{s}=C_{s}\left(1-e^{\left(-Pt\right)}\right) \end{array}$$

or

(8) $$\begin{array}{c} C=C_{s}\left(1-e^{\left(-(\left(DS\right)/\left(Vh\right))*t\right)}\right). \end{array}$$

Considering that the data we used reflect the drug release percentage over time and *C* = *m*/*V* = *P*∗*m*(total)/*V*  (*P* is the drug release percentage, and *m*(total) is the total drug amount), the equation can be further transformed into

(9) $$\begin{array}{c} P=\frac{V}{m\left(\text{total}\right)}*C_{s}*\left(1-e^{\left((\left(-DS\right)/\left(Vh\right))*t\right)}\right). \end{array}$$

While *P* ≥ 0 and *P* ≤ 1, *t* ≥ 0.

## 3. Results and Discussion

### 3.1. The Fourier Transform Infrared (FT-IR) Spectroscopy

**Figure 1:**
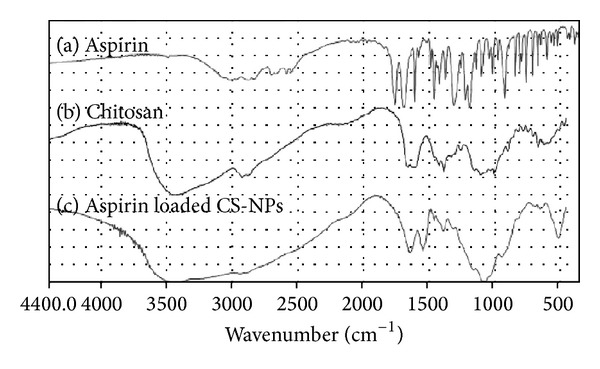
Transmission infrared spectra of aspirin, chitosan, and CS-NPs.

The transmission infrared spectra of aspirin loaded CS-NPs are shown in comparison with chitosan-free NPs and pure aspirin in Figure 1. Compared with chitosan free NPs and pure aspirin, the transmission infrared spectra of aspirin loaded CS-NPs is obviously different, explaining that aspirin have already been loaded to the CS-NPs.

### 3.2. Differential Scanning Calorimetry (DSC)

**Figure 2:**
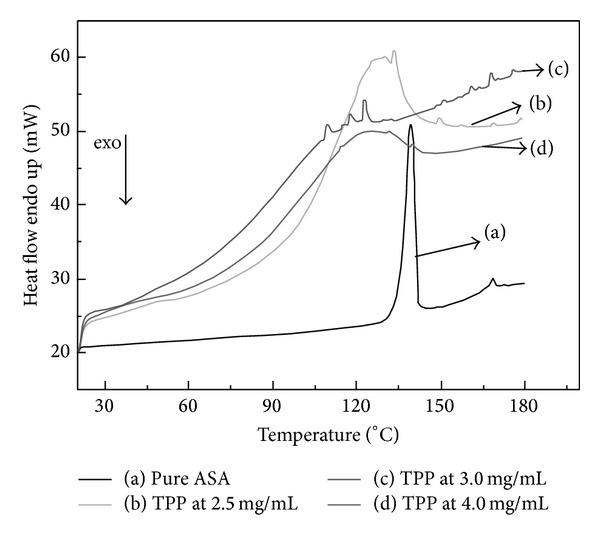
DSC thermograms of (a) pure aspirin and 0.8 mg/mL aspirin loaded CS-NPs with (a) pure ASA, (b) 2.5 mg/mL TPP, (c) 3.0 mg/mL TPP, and (d) 4.0 mg/mL TPP.

The DSC thermograms of aspirin and aspirin loaded CS-NPs with different TPP concentrations are shown in Figure 2. The melting peak of pure aspirin (Figure 2(a)) occurred at 139°C. A melting peak of aspirin loaded CS-NPs with 2.5 mg/mL TPP (Figure 2(b)) was found at 139°C which is due to the physical connection between aspirin and CS-NPs, and some little peaks were found during 150–160°C, which was probably due to the chemical connection between aspirin and CS-NPs. The thermogram of aspirin loaded CS-NPs with 3.0 mg/mL TPP (Figure 2(c)) showed some little peaks during 150–160°C and the melting peak of aspirin at 139°C was not present, confirming the aspirin in CS-NPs at a molecular level with 3.0 mg/mL TPP. A thermogram of aspirin loaded CS-NPs with 4.0 mg/mL TPP (Figure 2(d)) showed a clear melting peak at 139°C which is almost the same with Figure 2(b) and the little peaks during 150–160°C disappeared, indicating the physical connection between aspirin and CS-NPs.

### 3.3. Morphology Study

**Figure 3:**
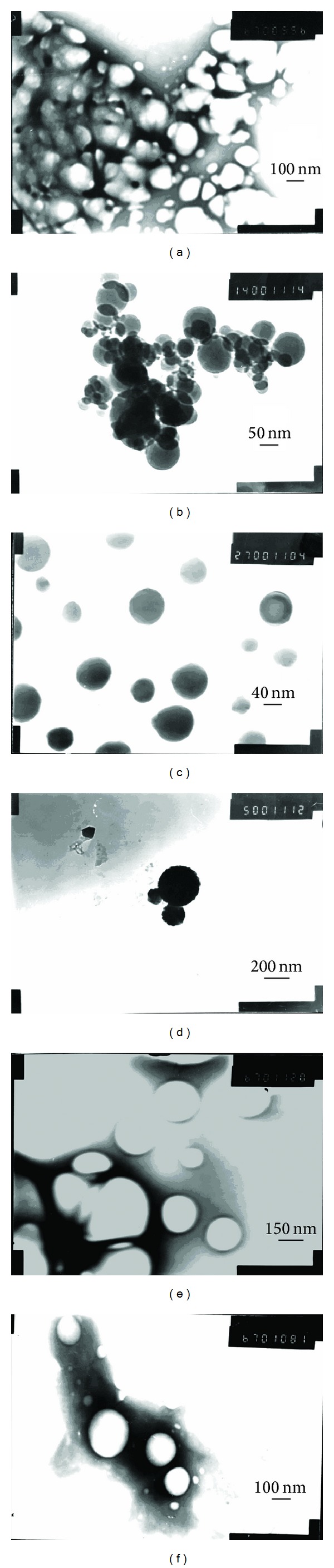
TEM of aspirin loaded CS-NPs ((a) ASA (0.4 mg/mL) and TPP (2.5 mg/mL); (b) ASA (0.8 mg/mL) and TPP (2.5 mg/mL); (c) ASA (1.2 mg/mL) and TPP (2.5 mg/mL); (d) ASA (0.4 mg/mL) and TPP (5.0 mg/mL); (e) ASA (0.8 mg/mL) and TPP (5.0 mg/mL); (f) ASA (1.2 mg/mL) and TPP (5.0 mg/mL)).

The transmission electron photomicrographs of aspirin loaded CS-NPs are illustrated in Figure 3. The results revealed that the morphology of the prepared NPs were spherical in shape with a smooth surface. But the particle size is not symmetrical in distribution, and some conglutination and congregation of NPs were found. Figure 3 shows that the particle size of CS-NPs was different with both concentrations of aspirin and TPP. Photos (a), (b), and (c) in Figure 3 show the morphology of NPs, which were prepared with the same TPP and different aspirin concentrations. It was found that the particle size decreased slightly with increasing aspirin concentration. The same phenomenon was found in photos (d), (e), and (f). It revealed that the concentration of aspirin has little influence on particle size of CS-NPs. By comparing photos (a) and (d), (b) and (e), and (c) and (f), it was observed that the particle size increased greatly with increasing TPP and constant aspirin concentration. It proved that the TPP concentration has great influence on particle size of CS-NPs.

### 3.4. Drug Loading Efficiency

#### 3.4.1. Effect of TPP Concentration

**Figure 4:**
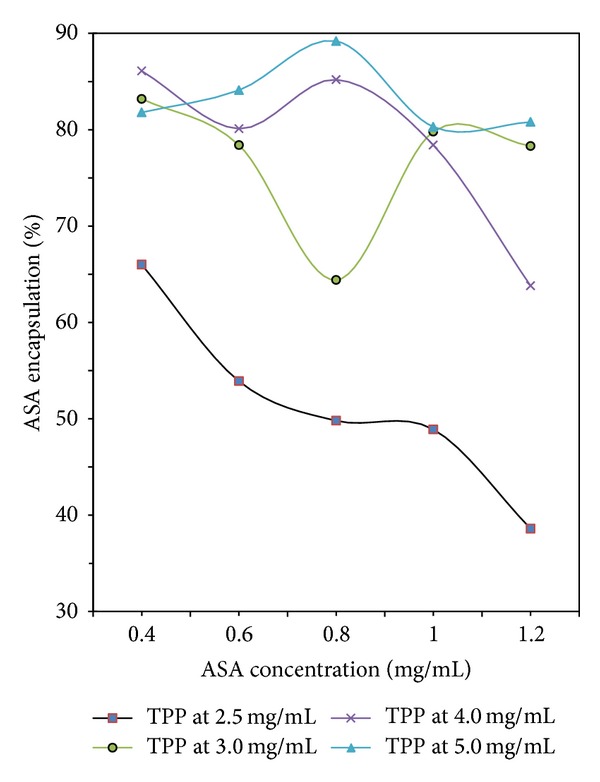
Aspirin encapsulation efficiency with different concentration of TPP and ASA.

**Figure 5:**
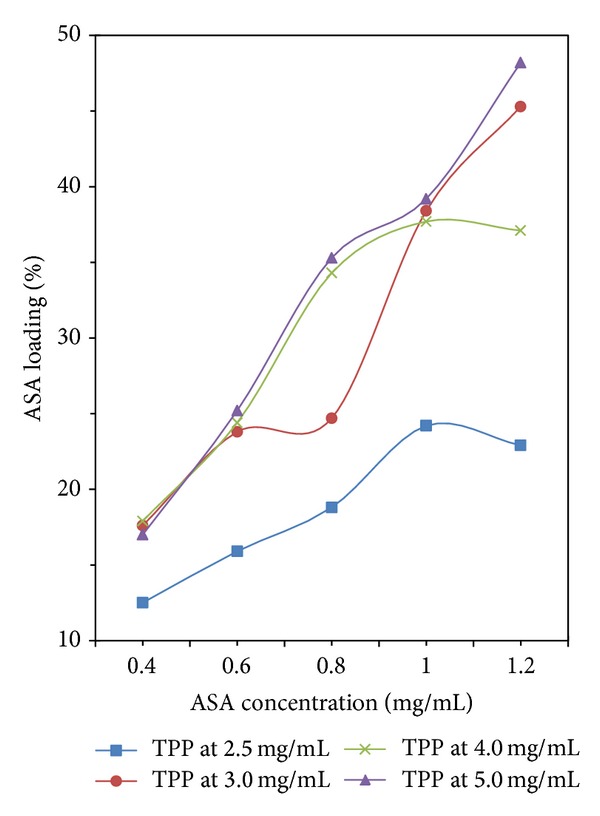
Aspirin loading capacity with different concentration of TPP and ASA.

The aspirin encapsulation efficiency from 37% to 90% was significantly affected by the TPP concentration in Figure 4, and the higher the concentration is, the higher the encapsulation efficiency is. Also, the loading capacity increased from 13% to 50% in general by increasing the TPP concentration from 2.5 to 5.0 mg/mL in Figure 5. The result indicated that the drug loading capacity of CS-NPs increased with increasing TPP concentration.

#### 3.4.2. Effect of Aspirin Concentration

As to different TPP concentration, the encapsulation efficiency and loading capacity of aspirin showed different variation trend in Figures 4 and 5. In Figure 4, the line of the TPP concentration = 2.5 mg/mL shows that increasing the initial aspirin concentration will decrease the encapsulation efficiency of aspirin. When the initial concentration of aspirin was 0.8 mg/mL, the line of the TPP concentration = 3.0 mg/mL shows the encapsulation efficiency is at the bottom, while both lines of the TPP concentration > 3.0 mg/mL show the encapsulation efficiencies are at the top. Moreover, the variation of loading capacity shown in Figure 5 was found to be similar to that shown in Figure 4.

#### 3.4.3. Effect of Chitosan Molecular Weight

**Figure 6:**
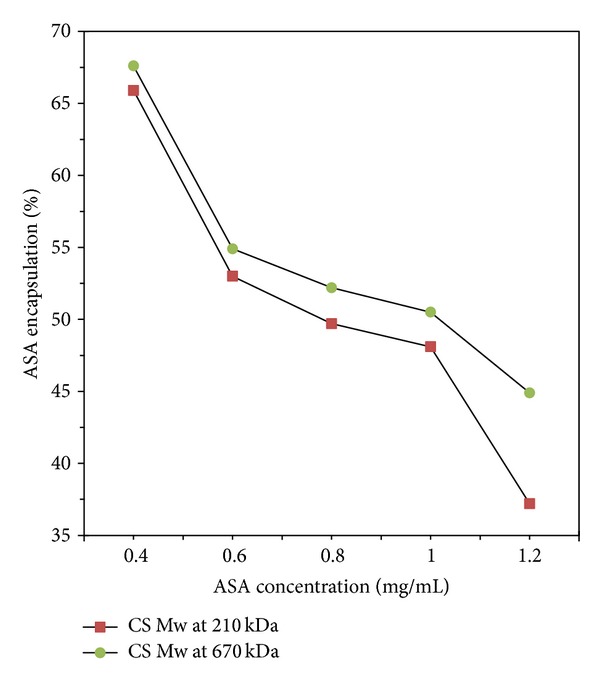
Aspirin encapsulation efficiency with different chitosan Mw.

**Figure 7:**
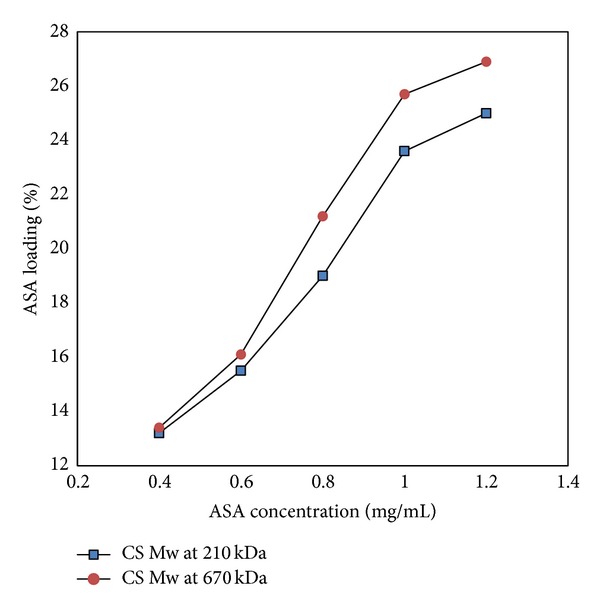
Aspirin loading capacity with different chitosan Mw.

As shown in Figures 6 and 7, the influence of chitosan Mw on the drug loading efficiency of aspirin was not significant when the concentration of aspirin was lower than 1.0 mg/mL. The encapsulation efficiency increased by almost 2% and the loading capacity increased by about 1% with increasing chitosan Mw. When the concentration of aspirin was higher than 1.0 mg/mL, the drug loading efficiency had a great increase with higher chitosan Mw. Increasing chitosan Mw up to 670 kDa made encapsulation efficiency and loading capacity increase by 7.5% and 5%, respectively. The TPP concentration used in this study is 2.5 mg/mL.

### 3.5. In Vitro Drug Release

**Figure 8:**
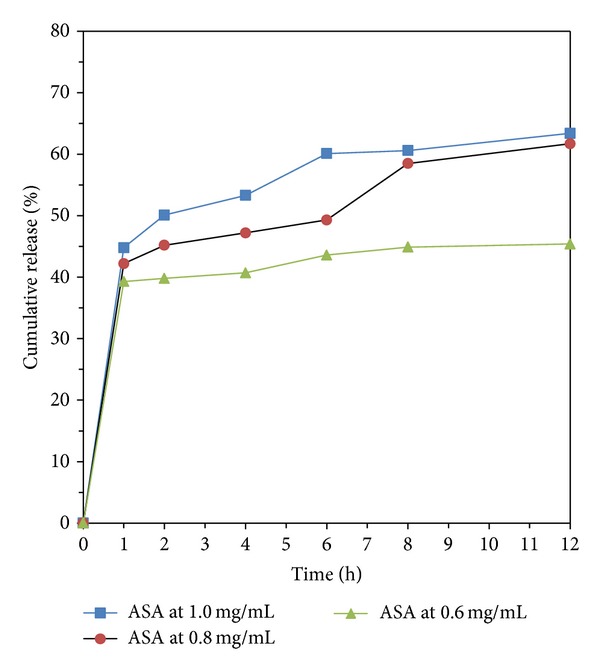
Drug release from CS-NPs when the TPP concentration is 2.5 mg/mL, with different initial aspirin concentration.

**Figure 9:**
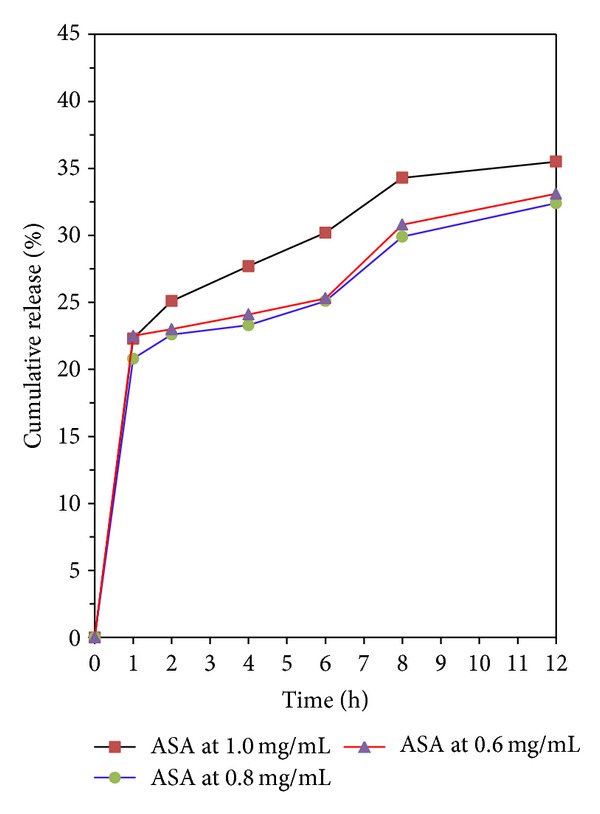
Drug release from CS-NPs when the TPP concentration is 3.0 mg/mL, with different initial aspirin concentration.

The aspirin in vitro release behavior of chitosan is shown in Figures 8-9. All release behavior lasted for more than 12 hours. It indicated that the CS-NPs showed a good performance of drug controlled release. Furthermore, all release profiles of the nanoparticles exhibit a small burst release in the first 1 h and then slow release at constant but different rate. The results suggest that it is possible to control the release rate of aspirin by adjusting the concentration of aspirin and molecular parameters of chitosan. In contrast, microsphere-based drug delivery system may have a higher percentage of burst release; therefore higher dosage is generally needed [35].

Moreover, we noticed that the drug release behavior with different TPP concentration is different when the initial aspirin concentration is 0.8 mg/mL. As shown in Figures 8-9, the line of ASA 1.0 mg/L and 0.8 mg/L showed a release plateau during 2 to 6 hours when the TPP concentration is lower than or equal to 3.0 mg/mL. As the TPP concentration is higher than 3.0 mg/mL, the release plateau disappeared as shown in Figure 9. Comparing the three lines of 1.0 mg/L to 0.6 mg/L to the other two lines (the aspirin concentration is 1.0 and 0.6 mg/mL), respectively, in Figures 8-9, we found that the sequence of the drug release speed is ASA 1.0 mg/L > ASA 0.6 mg/L > ASA 0.8 mg/L.

### 3.6. Mathematical Simulation Studies

Using the data in Figures 8-9, we find that the Noyes-Whitney equation approach can be used to interpret the original release curve. First, it explains why the release rate will be slowing down. With the increase of *t*, the growing rate of *e* ^(((−*DS*)/(*Vh*))∗*t*)^ will drop. Second, it explains why there is an upper limit for the final release percentage. It will approach zero when *t* is infinite, so *P* will reach its maximum value *V*/*m*(total)∗*C*_*s*_.

In addition, the formula also reflects some properties which are not exhibited in the experiment. The dissolution process may have two different end results. One is to approach certain maximum value as shown in the experiments, and the other is to continue the drug release process along the curve, eventually release completely so that *P* = 1. The key decision factor for the difference between these two results is the total dosage *m*(total). If the total dosage is small, *m*(total)/*V* < *C*_*s*_, and t is infinite, the maximum value of *P* = *V*/*m*(total)∗*C*_*s*_ is greater than 1 and the drug will be completely released. Otherwise, it approaches the maximum value. It is worth mentioning that *m*(total)/*V* is the concentration of the drug when it is fully released.

This formula also tells us when a drug is released inside the body, because of the very large amount of body fluid; that is, *V* is large; the value of *P* will soon reach its maximum. Therefore, the drug release profile in the body is mostly the first part of the experiment curve and is almost linear. If we want to control its speed, according to the equation, the measures we may take include reducing the surface area of the drug or changing the nature of the drug itself.

We also tested two other mathematical models, the probabilistic Boolean network model and the polynomial fitting. The initial purpose of designing the probabilistic Boolean network model [36, 37] is intended to be used in biological networks, in which, during biological activities, the changes among each state are dependent on certain factors. Its basic idea is the Markov chain; that is, the state in which the network will be at the next moment depends on the known states at this moment. Each of the network nodes represents a state of the actual biological events. The simulation results obtained from the probabilistic Boolean network model are not satisfactory. We first built a state transition table (not shown). According to the table, all the state transitions are stiff and determined (because all nonzero values are 1), which does not meet our expectations. There are two possible explanations for this. This model is most suitable for discrete states rather than continuous values and this model requires a large amount of raw data. Although we could forcibly discrete the continuous values, all the data are still concentrated in certain kind of state. Most of the positions are 0, and only a handful has nonzero values. Therefore, to improve the usefulness of the probabilistic Boolean network in the drug release studies, we may have to either further refine the data classification so that there are more differences that can be reflected among them or increase the amount of raw data.

Results for our polynomial fittings are not ideal either. Polynomial fitting is the modeling process in which a series of polynomial functions is used. It is based on the Taylor formula in which any kind of expression can be approximated by the sum of infinite polynomial functions, as shown below:

(10) f(x)=f(a)+f′(a)1!(x−a)+f(2)(a)2!(x−a)2+⋯+f(n)(a)n!(x−a)n+Rn(x).

Therefore, it is always possible to improve the fitting accuracy by adding more items. The purpose of the polynomial fitting is to get the relationship between the drug concentrations versus time. The first step is to determine using which mathematical formula to fit the individual data. We tried *C* = *t* ^*x*^, and to determine at what value of *x* the relationship between the drug concentration and time is most closely mirroring the experiment data. Our simulation (data not shown) indicates that, when the number of power is at 1.3 to 1.4 or so, the result is the closest to the experiment data. However, all the results for other power values are in fact close to experiment data too. And considering that this is just for one experiment, we cannot draw the conclusion that it is optimal. Even if this is indeed the numerically optimal solution, it may not be able to explain the mathematical laws behind this physical process. Therefore, the conclusions obtained by this method are not very convincing. To improve the credibility of polynomial fitting, we need a lot of data to improve. Even so, it cannot solve a more fundamental problem. Is this the scientific way? Does it reflect the mathematical relationship behind the physical processes? Therefore, to use this modeling method, we not only need a lot of data to derive an appropriate equation of the fitting form but also need data to validate the model.

## 4. Conclusions

The drug-polymer delivery system of aspirin/CS-NPs exhibits well sustained release performance. Encapsulation efficiency, loading capacity, thermal behavior, and drug release behavior of aspirin loaded CS-NPs were affected by TPP and aspirin concentration as well as the molecular weight of chitosan. Physicochemical characterization of all CS-NPs loaded with aspirin could reveal the drug physical state and drug-polymer interaction. The data of DSC demonstrated the drug-polymer interaction between aspirin and CS-NPs. The drug-polymer interaction affected the release of aspirin from the CS-NPs resulting in sustained release action. It was summarized that chitosan could interact with negatively charged drugs when encapsulated into CS-NPs. We also use mathematical models to simulate the drug release process, and the Noyes-Whitney equation approach is successful and can well explain the experiment data we obtained. This may provide us with a more convenient way to validate our data and predict experiment results in the future studies.
